# Impact of sunitinib resistance on clear cell renal cell carcinoma therapeutic sensitivity *in vitro*

**DOI:** 10.1080/15384101.2024.2306760

**Published:** 2024-01-23

**Authors:** Susmita Ghosh, Mamatha Garige, Patrick R. Haggerty, Alexis Norris, Chao-Kai Chou, Wells W. Wu, Rong-Fong Shen, Carole Sourbier

**Affiliations:** a Division of Biotechnology Review and Research 1, Office of Biotechnology Products, Office of Pharmaceutical Quality, Center for Drug Evaluation and Research, U.S. Food and Drug Administration, Silver Spring, MD, USA; b Division of Animal Bioengineering and Cellular Therapies, Office of New Animal Drug Evaluation, Center for Veterinary Medicine, U.S. Food and Drug Administration, Rockville, MD, USA; c Facility for Biotechnology Resources, Center for Biologicals Evaluation and Research, U.S. Food and Drug Administration, Silver Spring, MD, USA

**Keywords:** Sunitinib, RCC, PD-L1, OXPHOS, metformin, Axl

## Abstract

Sunitinib resistance creates a major clinical challenge for the treatment of advanced clear cell renal cell carcinoma (ccRCC) and functional and metabolic changes linked to sunitinib resistance are not fully understood. We sought to characterize the molecular and metabolic changes induced by the development of sunitinib resistance in ccRCC by developing and characterizing two human ccRCC cell lines resistant to sunitinib. Consistent with the literature, sunitinib-resistant ccRCC cell lines presented an aberrant overexpression of Axl and PD-L1, as well as a metabolic rewiring characterized by enhanced OXPHOS and glutamine metabolism. Therapeutic challenges of sunitinib-resistant ccRCC cell lines in vitro using small molecule inhibitors targeting Axl, AMPK and p38, as well as using PD-L1 blocking therapeutic antibodies, showed limited CTL-mediated cytotoxicity in a co-culture model. However, the AMPK activator metformin appears to sensitize the effect of PD-L1 blocking therapeutic antibodies and to enhance CTLs’ cytotoxic effects on ccRCC cells. These effects were not broadly observed with the Axl and the p38 inhibitors. Taken together, these data suggest that targeting certain pathways aberrantly activated by sunitinib resistance such as the AMPK/PDL1 axis might sensitize ccRCC to immunotherapies as a second-line therapeutic approach.

## Introduction

Clear cell renal cell carcinoma (ccRCC) is the most common type of kidney tumor and is characterized in 75% of the cases by an inactivation or mutation of the tumor suppressor gene *von Hippel Lindau* (*VHL*). VHL inactivation leads to the aberrant stabilization and activation of the hypoxia inducible factor (HIF) which creates a pseudo-hypoxic state [1]. Numerous efforts to target the aberrant activation of the HIF pathway have been made and the use of anti-angiogenic therapies such as sunitinib or sorafenib, and of paralogues, such as temsirolimus, have shown some clinical benefits. Sunitinib has been approved as first-line therapy for patients with advanced ccRCC over a decade ago; however, patients who received sunitinib therapy gradually develop resistance after several months. In recent years, immune check point inhibitors (ICIs) have been approved as second-line therapies for patients with sunitinib-resistant ccRCC, as well as first-line therapy. It is, however, unclear if and how sunitinib resistance affects ICIs’ efficacy.

Tyrosine kinase receptors (RTKs), including vascular endothelial growth factor receptors (VEGFRs) and their ligands, play important roles in tumor growth and angiogenesis. Inhibition of VEGF signaling using VEGF blocking antibodies or VEGFR antagonists has demonstrated potent antitumour effects [2–6]. Sunitinib (sunitinib malate) is a multi-targeted tyrosine kinase inhibitor targeting VEGFR-1, VEGFR-2, fetal liver tyrosine kinase receptor 3 (FLT3), KIT (stem-cell factor [SCF] receptor), PDGFRα, and PDGFRβ [7,8]. The development of sunitinib resistance has been a clinical challenge, leading to numerous studies aiming to understand how to overcome it [9]. Several signaling pathways have been identified as key players in the development of sunitinib resistance in ccRCC. For example, Axl, a member of the family of TAM receptors, is an essential mediator of cancer metastasis [10] and is known to be aberrantly expressed and activated in sunitinib resistant ccRCC cell lines [11,12]. Other pathways aberrantly activated or modulated by the development of sunitinib resistance in ccRCC and other tumors include the p38 MAP kinase [13] as well as the AMPK/PD-L1 axis. HSP27 is a downstream effector of p38 MAP kinase [14] and increased HSP27 phosphorylation has been observed in renal cancers, as well as in other kidney diseases [15,16]. SB203580, an inhibitor of Ser/Thr kinase, p38 MAP kinase, increases the phosphorylation of p38 MAP kinase and decreased the phosphorylation of HSP27 [17,18]. Sunitinib-resistant ccRCC cells display low AMPK activation and elevated PD-L1 expression [19,20]. Recent studies have shown that metformin-activated AMPK directly binds to and phosphorylates PD-L1 on Serine195, which leads to its degradation [21]. In brief, bemcentinib, SB203580, and metformin have all been suggested as potential therapeutic agents for sunitinib-resistant ccRCC.

We have recently shown that PD-L1 expression is required to mediate some of IFNγ’s effect in ccRCC cells in a ligand-independent manner, highlighting the importance of PD-L1 signaling in regulating the metabolism of ccRCC cells in response to inflammatory signals [22]. Changes in cellular metabolism and signaling pathways induced by sunitinib resistance might therefore modulate the ccRCC tumor microenvironment. The metabolism of ccRCC is flexible and tends to shift toward aerobic glycolysis with grade [23]. Another driver of metabolic flexibility and adaptation is therapeutic pressure. By targeting the vasculature and the tumor’s access to oxygen and nutrients, TKIs such as sunitinib directly and indirectly affect ccRCC tumor’s hypoxic environment, and hence their metabolism. Therefore, understanding how TKIs reshape ccRCC metabolism is a first necessary step to predict the efficacy of second-line therapies such as immune checkpoint inhibitors (ICIs) by potentially using multi-omics approaches [24,25].

ICIs have recently changed the therapeutic landscape of ccRCC [26–28]. Several ICIs have been approved by the US FDA over the last four years for the treatment of advanced ccRCC, and more than 25 current clinical trials are including at least one ICI [26,29]. With this expansion of ICIs use, it is critical to improve our understanding of their mechanism of action and to develop tools to identify who will benefit from these therapies. Use of ICIs as first- and second-line therapy for patients with advanced ccRCC is expanding. Under-standing how first-line therapies such as TKIs reshape ccRCC metabolism will significantly improve our understanding of TKIs and ICIs mechanism of action, which will allow for the development of tools to identify safe and effective second-line therapies. Thus, the goals of this projects were to develop and characterize an RCC model of resistance to sunitinib and to evaluate what could be potential therapeutic approaches for these tumors.

## Materials and methods

### Cell lines and cell culture

Two human advanced renal cell carcinoma cell lines A498 and 786-O were purchased from American Type Culture Collection (ATCC; Manassas, VA). They were cultured in Dulbecco’s Modified Eagle’s Medium (DMEM) growth media containing 25 mM glucose and glutamine supplemented with 10% heat-inactivated fetal bovine serum and 1% penicillin-streptomycin at 37°C in a humidified atmosphere of 5% CO2.

### Development of sunitinib-resistant RCC cell lines

Sunitinib (SU11248) malate was purchased from Selleckchem (Houston, TX). For the development of sunitinib-resistant cells, parental cells were exposed to increasing concentrations of sunitinib. In brief, the RCC cell lines were treated with varying concentrations of sunitinib (0, 1, 2, 3, 4, 5, 7, 10, 20 and 50 μM). With the passage of time of every 4 days, it has been observed that A498 cells showed stable growth and eventually became confluent at a concentration of 2 µM, while 798-O cells showed stable growth to confluence at a concentration of 4 µM. It can be assumed that cells that grew to confluence had developed stable sunitinib-resistance after a period of 4 months or > 20 passages. The cells were constantly in sunitinib containing medium throughout the selection process.

### Drug treatment

Sunitinib resistant cells were treated with Axl inhibitor R428 (Selleckchem; Houston, TX), p38 MAPK inhibitor, SB203580 (Selleckchem; Houston, TX) and AMPK activator, Metformin hydrochloride (Sigma; St. Louis, MO). R428 and SB203580 were dissolved in DMSO and stored as 10 mM stock at −20°C. Metformin was dissolved in H2O and stored as 50 mM stock at −20°C.

### Cell viability assay

Cells were seeded in 96 well plates (5 × 103 cells/well/100 μL) and cell viability assay was performed using the CellTiter-Glo Luminescent Cell viability assay kit (Promega; Madi-son, WI) after treatment of the cells with a concentration-range of sunitinib 48hrs. The percentage of cell viability was calculated relative to the control wells, which were designated as 100%.

### RNA extraction

RNA was isolated from cultured cells according to the QIAGEN RNeasy mini kit protocol. RNA quality and quantity were assessed using a NanoDrop (Thermo Fisher).

### mRNA sequencing and differential gene expression analysis

RNA sequencing analysis was performed as previously described [22]. Briefly, the instrument used paired-end sequencing (100×2cycles) of multiplexed mRNA libraries and was carried out on Illumina NovaSeq 6000 and HiSeq 2000 sequencers (Illumina Inc., San Diego, CA). Libraries were prepared using the TruSeq Stranded mRNA Library Sample prep kit (Illumina Inc., San Diego, CA, USA). Sequencing reads were trimmed using trimmomatic (version 0.36.6; parameters: SLIDINGWINDOW:4:20 MINLEN:50) and then aligned to GRCh38/hg38 using HISAT2 (version 2.1.0; parameters: –n-ceil L,0.0,0.15 –mp 6,2 –no-softclip – np 1 –rdg 5,3 –rfg 5,3 –sp 2,1 –score-min L,0.0,-0.2 –pen-cansplice 0 –pen-noncansplice 12–pen-canintronlen G,-8.0,1.0–pen-noncanintronlen G,-8.0,1.0 –min-intronlen 20 –max-intronlen 500,000). Gene counts were estimated using featureCounts (version 1.6.3; parameters: -s 1 -t “exon” -g “gene_id” -J -Q 12 –minOverlap 1 –fracOverlap 0 –fracOverlapFeature 0) with GENCODE gene annotation (version 33; Ensembl version 99). To determine differential expression for genes of interest, we performed two-sided Welch Two Sample t-test of gene counts with *p* < 0.05 significance threshold.

Raw data are available on the GEO repository (ID# GSE216494).

### Protein extraction and Western blot assay

Cell pellets were lysed in radioimmune precipitation assay (RIPA) buffer containing 50 mM Tris (pH 7.4), 150 mM NaCl, 1% NP40, 0.25% sodium deoxycholate, 0.1% SDS, 1 × Protease inhibitor cocktail set 1 (Calbiochem; La Jolla, CA). Protein concentration was determined with the bicinchoninic acid BCA assay (Pierce from Thermo Fisher Scientific; Waltham, MA), and equal amounts of proteins (15 μg) were separated by NuPAGE®Novex® 4–12% Bis-Tris protein gels using NuPAGE®MES SDS Running Buffer (Life Technologies; Grand Island, NY). Proteins then were transferred onto 0.2 μm PVDF membranes (Invitrogen; Waltham, MA), which were subsequently blocked with 5% nonfat milk for 1 h and probed with the indicated antibodies (AXL, 1:1000, CST; pAXL, 1:1000, CST; pSTAT3, 1:1000; STAT3, 1:1000; pERK, 1:1000, CST; ERK, 1:1000, CST; pAKT, 1:1000, CST; AKT, 1:1000, CST; Actin 1:3000, CST; PD-L1, 1:1000, Abcam, HSP27, 1:1000, CST; pHSP27, 1:1000, CST; AMPK, 1:1000, CST; pAMPK, 1:1000, CST), and then incubated with corresponding HRP-linked secondary antibody (1:2000, CST, CA, USA) at 4°C over-night. Membranes were then washed, and blots were developed with the enhanced western Chemiluminescent HRP Substrate (Pierce from Thermo Fisher Scientific; Waltham, MA) for 2–3 min, and then photographed using a Bio-Rad Gel Doc XR and Imaging System.

### Measurement of cellular mitochondrial respiration

Cells at a density of 0.5 × 104 cells/well were seeded in 96-wells of an Agilent Seahorse XF96/XFe96 V3 PS Cell Culture Microplate (Agilent; Santa Clara, CA) until confluence (about 48 hrs). Experiments were performed in triplicate with *N* = 6 wells/treatments group. Oxygen consumption rates (OCR) and extracellular acidification rates (ECAR) were measured according to Seahorse XFp Cell Mito Stress Test Kit protocols recommended by Agilent. Briefly, cells were equilibrated in XF base medium without phenol red containing 10 mM glucose, 2 mM-glutamine, and 1 mM sodium pyruvate. Cells were treated with oligomycin, FCCP, and a mixture of antimycin A and rotenone via injection ports as recommended in the manufacturer’s protocol to measure mitochondrial basal respiration, ATP-linked respiration, H+ (proton) leak, maximal respiration, spare respiratory capacity, and non-mitochondrial respiration baseline OCR and ECAR using the Agilent Seahorse XF96/XFe96 bioanalyzer. Data presented are normalized to cell number per well as estimated by CyQUANT staining (Thermo Fisher Scientific; Waltham, MA).

### Lentivirus transduction and generation of stable cell lines

Stable Renilla luciferase expressing cell lines were generated as previously described [22] by lentiviral transduction using LP462–025 lentiviral particles (Genecopia; Rockville, Maryland) with the concentration of lentivirus at MOI (multiplicity of infection) 5.0 in the presence of 5 μg/mL Polybrene (Sigma; St. Louis, MO) for 24 hrs. Cells with stable expression of Renilla luciferase were cultured in media containing 0.5–5 mg/ml puromycin. Renilla luciferase expressing 786-O parental cells and 786-O sunitinib resistant stable cells were selected from the cells that were growing in 3 μg/ml and 0.5 μg/ml puromycin, respectively, whereas Renilla luciferase expressing A498 parental cells and A498-Su sunitinib resistant stable cells were selected from the cells that were growing in 3 μg/mL and 1 μg/ml puromycin respectively.

### Co-culture with Cytotoxic T Lymphocytes (CTLs)

Renilla luciferase expressing sunitinib resistant stable cells (1.5 × 104/well) were seeded in 96-well plate 2 days in advance and then co-cultured with CTLs for 2–3 days. Human peripheral blood CD8+T cells (CTLs) were purchased from StemCell Technology Inc (Vancouver, Canada) and cultured in RPMI media supplemented with 10% FBS, 1% penicillin/streptomycin and 0.1 mg/mL hIL2. They were activated with ImmunoCult™ Human CD3/CD28 T cell Activator (StemCell Technology Inc., Vancouver, Canada) and cultured in a humidified incubator containing 5% CO2 at 37°C for 3–4 days before co-culture with cancer cells. The ratio of cancer cells and CTLs was optimized to 1:2 to 1:4. Sunitinib resistant cells were co-incubated with CTLs alone or CTLs with R428, SB203580 and Metformin at desired concentrations for indicated time. After incubation, luciferase luminescence was evaluated using Renilla-Glo Luciferase assay kit (Promega; Madison, WI) to estimate cancer cell viability. Caspase-Glo 3/7 assay (Promega; Madison, WI) was used to evaluate cancer cell caspase activities or cell death in duplicate plates parallelly.

## Results

### Development and characterization of two sunitinib-resistant ccRCC cell lines in vitro

**Figure 1.**
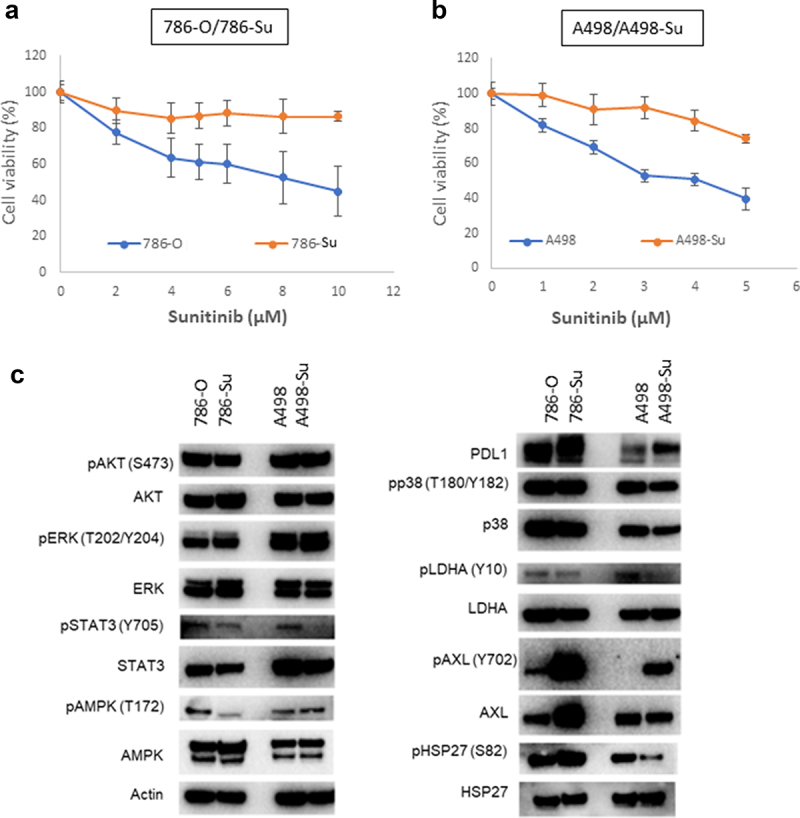
Development of sunitinib-resistant ccRCC cell lines. cell viability assay following sunitinib treatment shows that 786-su (a) and A498-su (b) are resistant to sunitinib compared to naïve cells; (c) immunoblotting for key members of signaling pathways known to be activated following the development of sunitinib resistance.

Two sunitinib-resistant ccRCC cell lines were established by exposing 786-O and A498 cell lines to sunitinib for 4 months or > 20 passages (786-Su: 4 μM sunitinib; A498-Su: 2 μM sunitinib). Sensitivity of 786-Su and A498-Su to sunitinib was assessed by cell viability using a CellTiter-Glo luminescent cell viability assay kit (Promega; Madison, WI) after 48 hrs of treatment with a concentration-range of sunitinib. As shown in (Figure 1(a,b)), sunitinib minimally affected 786-Su and A498-Su survival even at high concentrations, while 786-O and A498 viability decreased in a dose-concentration manner. These data confirm the resistance of 786-Su and A498-Su toward sunitinib. Next, the alteration of signaling pathways associated with sunitinib-resistance was evaluated by immunoblot-ting, comparing the parental cell lines 786-O and A498 to 786-Su and A498-Su. Compared to 786-O and A498, sunitinib-resistant ccRCC cell lines 786-Su and A498-Su presented an aberrant overexpression of Axl and PD-L1, as well as a decreased phosphorylation of members of the STAT3 pathway and of LDHA (Figure 1(c)). This dysregulation of both the JAK/STAT and Axl pathways in sunitinib-resistant ccRCC cells is consistent with the literature [30]. In addition, the increase in PD-L1 expression and decrease in the phosphorylation of LDHA suggest that, if similar changes occur in vivo, sunitinib resistance may affect the ccRCC tumor microenvironment by becoming more immunosuppressive and less glycolytic.

**Figure 2.**
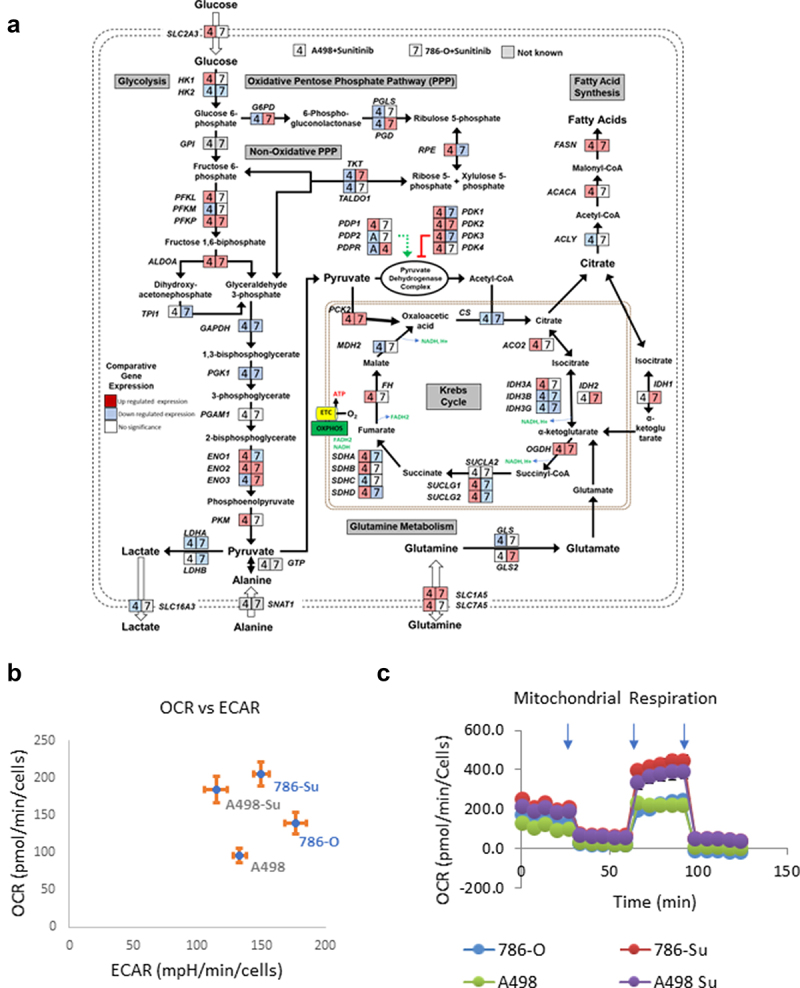
Metabolic characterization of sunitinib-resistant ccRCC cell lines. (a) mapping of the transcriptional regulation of selected metabolic enzymes following development of sunitinib resistance in 786-O and A498; (b) basal oxygen consumption rates (OCR) and extra-cellular acidification rates (ECAR) of 786-O, A498, 786-su and A498-Su; (c) OCR following mitochondrial stress injection of oligomycin, FCCP, and Rotenone/Antimycin). Refer to supplemental Figure 1 for data regarding spare capacity and ATP production.

Next, we performed mRNA sequencing analysis to further assess how 786-Su and A498-Su differ from A498 and 786-O (GSE216494). The expression levels of the metabolic gene’s transcripts were mapped into a metabolic pathway network (Figure 2(a)), representing the transcripts significantly either up- or downregulated in both cell lines after developing resistance. 786-Su and A498-Su presented a transcriptional pattern consistent with a general cellular metabolic shift toward glutamine and lipid metabolism and a decrease in aerobic glycolysis (Figure 2(a)). These data were confirmed in vitro using a seahorse bioanalyzer to measure the cellular respirations and extra-cellular acidification rates (a surrogate for lactate secretion, thus for glycolysis) of A498-Su, 786-Su as well as A498 and 786-O. Compared to parental 786-O and A498, sunitinib-resistant A498-Su and 786-Su cells presented an increased oxygen consumption rate (OCR) while their extracellular acidification rates (ECAR) were decreased (Figure 2(b,c)), suggesting that the metabolism of the cells resistant to sunitinib had shifted toward oxidative phosphorylation. A498-Su and 786-Su cells also appeared more metabolically active and flexible than parental A498 and 786-O cells, with higher spare respiratory capacity and higher ATP consumption than their nonresistant counterparts (Supplemental Figure 1). Taken together, these data confirmed the metabolic shift of sunitinib-resistant cell lines toward oxidative phosphorylation, which is consistent with the literature [31,32].

### Targeting of signalling pathways aberrantly regulated in sunitinib-resistant ccRCC cell lines

**Figure 3.**
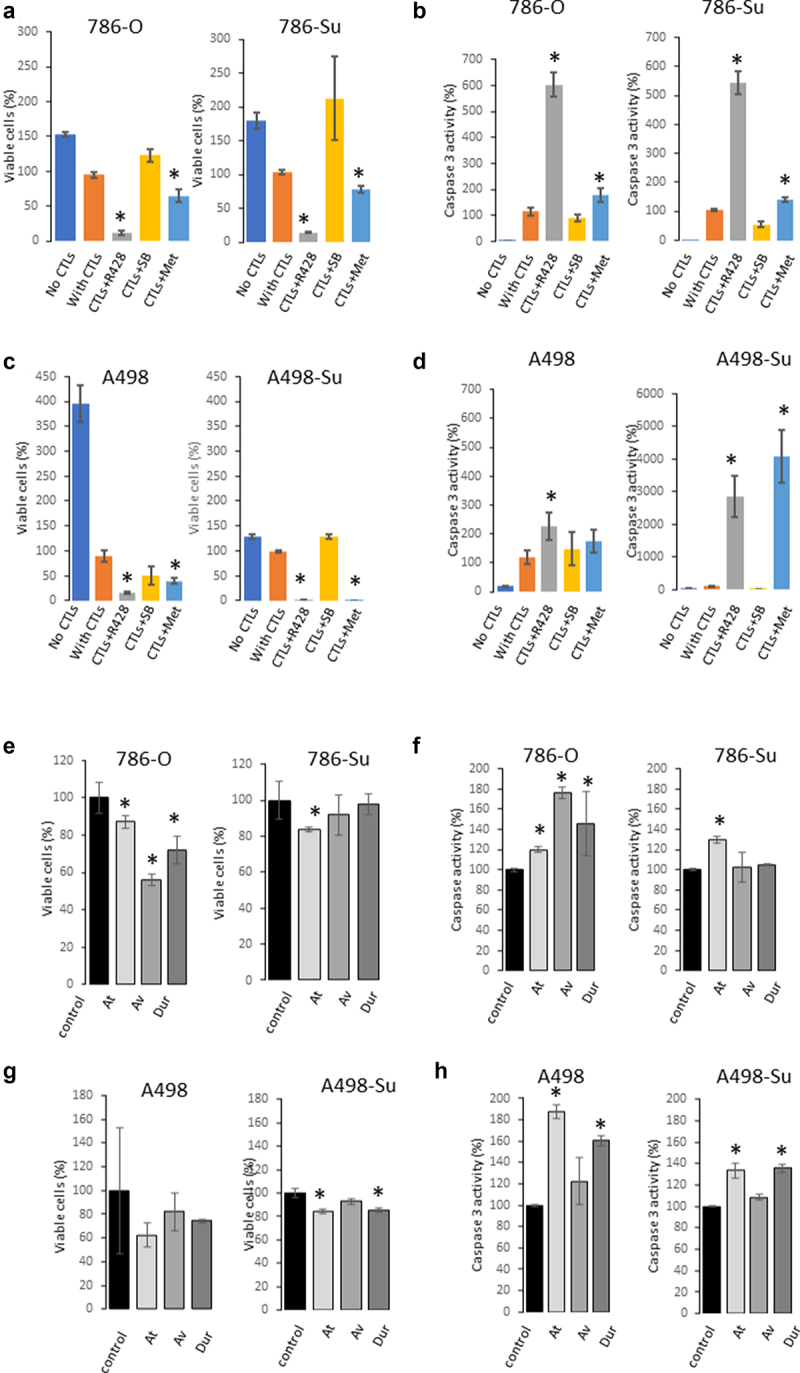
Viability of 786-su and A498-su following different treatments. cell viability (a) and caspase 3 activity (b) following treatment with axl inhibitor (R498), MAPK inhibitor (SB203835) and AMPK activator (metformin) in 786-O and 786-su cells. Cells were treated for 24 hours. Cell viability (c) and caspase 3 activity (d) following treatment with axl inhibitor (R498), MAPK inhibitor (SB203835) and AMPK activator (metformin) in A498 and A498-su cells. Cell viability (e) and caspase activity (f) in 786-O and 786-su in a co-culture experiment with cytotoxic T cells using 3 PD-L1 blocking antibodies (20 mg/mL; IFNγ; avelumab, atezolizumab, and durvalumab). Cell viability (g) and caspase activity (h) in A498 and A498-su in a co-culture experiment with cytotoxic T cells using 3 PD-L1 blocking antibodies (20 mg/mL; IFNγ; avelumab, atezolizumab, and durvalumab).

Aberrant overexpression of PD-L1 and of the Axl and MAPK pathways was observed at the protein level in sunitinib-resistant cell lines. Because of the significant use of immune checkpoint inhibitors for the treatment of ccRCC patients, we investigated whether modulation of Axl, PD-L1 and MAPK/HSP27 may affect the response of sunitinib-resistant ccRCC cell lines to PD-L1 blocking therapeutic antibodies. We compared the efficacy of these three targeting agents in vitro between nonresistant and sunitinib-resistant ccRCC cell lines. 786-Su and A498-Su cells were treated with the Axl inhibitor R428, the AMPK activator metformin, and the MAPK inhibitor SB203580 while in co-culture with cytotoxic T cells (CTLs; Figure 3). R428 is a direct Axl inhibitor that has been shown to enhance anti-PD1 therapies in nonclinical models of solid tumors [33]. The AMPK activator metformin has been shown to mediate the phosphorylation of PD-L1, which leads to its degradation and decrease in PD-L1 protein expression [21]. SB203580 is a p38 and Akt inhibitor that is known to induce HSP27 phosphorylation leading to its in-activation [34]. HSP27 is also downstream of the MAPK pathway. As shown in (Figure 3(a-d)), R428 and metformin supported the effects of CTLs in resistant and nonresistant cell lines, while SB203580 did not present any significant effect. An increase in caspase activity paralleled the decrease in cell viability, indicating that the decrease in viability was due to apoptosis.

The mechanism of action of PD-L1 blocking therapeutic antibodies is to block the interaction between PD1 on CTLs and PD-L1 on tumor cells. Using a co-culture in vitro model, we assessed the cytotoxic effect of CTLs on sunitinib-resistant and naïve ccRCC cell lines following treatment with 3 anti-PD-L1 blocking therapeutic antibodies (avelumab, atezolizumab, durvalumab). As shown in (Figure 3(e-h)), in concordance with our recently published paper [22], treatment of cells with CTLs at 1:4 ratio, at 20 mg/mL of ICIs for 24 hours in the presence of IFNγ significantly decreased the viability of 786-O and A498 cells while increasing caspase activity. Consistent with the observed increase in PD-L1 expression, the cytotoxic effect of CTLs in sunitinib-resistant cell lines A498-Su and 786-Su was decreased. These data suggest that in this model of sunitinib resistance, ccRCC sunitinib-resistant cells may be less sensitive to ICIs therapies.

**Figure 4.**
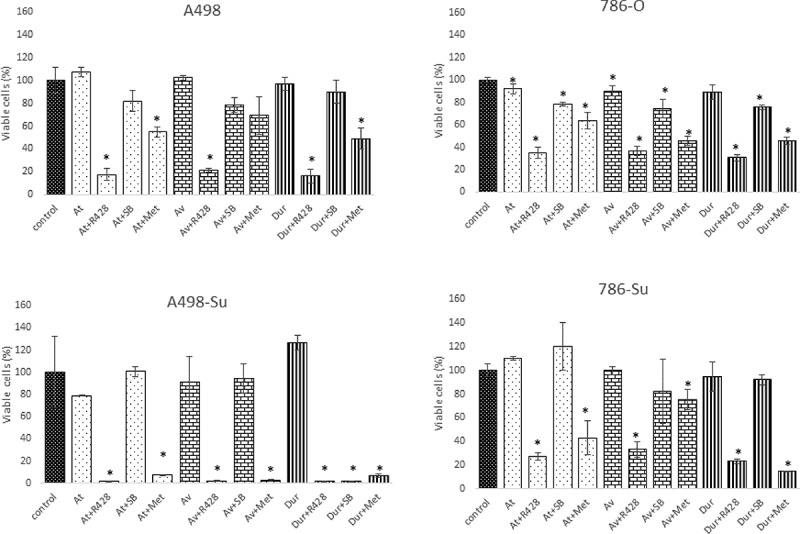
Cell viability of 786-O and 786-su and A498 and A498-su in a co-culture experiment with cytotoxic T cells following treatment with 3 PD-L1 blocking antibodies (10 mg/mL; 24 hours), combined with Axl inhibitor (R498), MAPK inhibitor (SB203835) and AMPK activator (metformin). *: *p* < 0.05.

Next, we assessed whether combining PD-L1 therapeutic antibodies with either the Axl inhibitor R428, AMPK activator metformin, or MAPK inhibitor SB203580 might have a therapeutic value. We performed another co-culture experiment with CTLs at 1:2 ratio, ICIs at 10 mg/ml (absence of IFNγ) for 24 hours with the different agents (R428, SB203580 and metformin). As shown in (Figure 4(a,b)), in 786-O and A498 cells, the MAPK inhibitor SB203580 and the Axl inhibitor R428 did not show any significant additive effect in the co-culture model, while Metformin presented a mild effect. In sunitinib-resistant cell lines 786-Su and A498-Su, Metformin strongly enhanced the CTLs cytotoxic effects in both cell lines, and R428 and SB203580 presented a strong effect, although not consistently across the ICIs used (Figure 4(c,d)). For example, SB203580 only enhanced the effect of durvalumab in A498-Su and R428 enhanced the sensitivity of the 3 ICIs in A498-Su but not in 786-Su. Although the 3 ICIs have a similar target (PD-L1), they might exert different effects on the tumor cells, potentially because of differences in the monoclonal antibodies’ post-translational modifications such as glycosylation [35,36]. Taken together, these data demonstrated that combining metformin with PD-L1 blocking therapeutic antibodies enhanced CTLs efficacy against sunitinib-resistant ccRCC cell lines using a co-culture in-vitro model. This could potentially be a valuable therapeutic approach for ccRCC patients who developed a resistance to sunitinib, although further nonclinical and clinical investigations would be necessary to confirm these findings.

## Discussion

Clear cell renal cell carcinoma (ccRCC) is the most common type of kidney cancer, accounting for 75–85% of all renal cell carcinoma patients and more than 100,000 deaths every year. Sunitinib is a standard first-line treatment for metastatic ccRCC; however, the clinical benefit of sunitinib on progression-free-survival is limited, as more than half of patients do not respond to initial therapy, and those who do, are likely to develop resistance after ~24 months [37]. Therefore, there is a need to better understand the effect of the development of sunitinib resistance on the physiology of ccRCC to identify potential therapeutic approaches. Although multiple publications have investigated the molecular basis of sunitinib resistance [38–40], unknowns remain about which second-line therapies to select and how sunitinib resistance may affect immunotherapy efficacy. In this project, we investigated how sunitinib resistance affected the transcriptional profile of ccRCC cell lines and based on these findings, explored some therapeutic approaches.

We generated two sunitinib-resistant clones (786-Su and A498-Su) from commercially available ccRCC cell lines that are deficient in VHL (786-O and A498) and characterized the biological and metabolic changes induced by sunitinib resistance. Immunoblotting revealed that sunitinib resistance-induced overexpression of PD-L1 and Axl as well as decreased phosphorylation of STAT3 and LDHA (Figure 1), suggesting a metabolic rewiring of the sunitinib-resistant cell lines. To further assess the metabolic changes that occurred in 786-Su and A498-Su, we mapped metabolic genes’ expression patterns into a concrete metabolic pathway network and profiled the energetic processes of these cell lines using a seahorse bioanalyzer (Figure 2). 786-Su and A498-Su presented a general cellular metabolic shift toward glutamine and lipid metabolism, as well as increase in ATP production and spare respiratory capacities, which together suggest that 786-Su and A498-Su have become more metabolically active and potentially aggressive than their parental counterpart 786-O and A498 cells. Multiple metabolomics and multi-omics studies have shown that ccRCC are characterized by a reprogramming of energetic metabolism, including glucose, lipid and glutamine metabolisms [23,41–46]. Our data are consistent with these studies as well as with metabonomic studies of ccRCC that enhanced glutamine metabolism was related to sunitinib resistance, with the glutamine transporter SLC1A5 being significantly overexpressed in sunitinib-resistant samples compared to the control group [32]. In addition, in a ccRCC primary culture model, fatty acid oxidation could be activated based on the metabolic needs of the cells, further supporting the idea that the observed shift toward lipid metabolism after development of sunitinib resistance might be reflective of the metabolically active phenotype of the cells [41].

Next, using a co-culture in vitro model using CTLs and ccRCC cell lines (Figures 3 and 4), we investigated the effects of several small molecule inhibitors targeting signaling pathways that are aberrantly regulated in sunitinib-resistant cell lines and that have been shown to affect the sensitivity of PD-1/PD-L1 therapeutic approaches in other nonclinical models [47–49]. R428, an Axl inhibitor, metformin, an AMPK activator, and SB203580, a p38 inhibitor were selected. SB203580 did not affect the sensitivity of the PD-L1 blocking antibodies in our model. One potential explanation of this lack of effectiveness may be due to the triggering of autophagy, similarly to what was described by Grossi and collaborators in prostate cancer [50]. Both R428 and metformin treatments significantly enhanced the effect of PD-L1 blocking antibodies in activating CTLs against sunitinib-resistant cell lines, with the effect of metformin being greater. Metformin is known to block gluconeogenesis through specific targeting of the mitochondrial isoform of GPDH [51]. Secondary to these effects, AMPK activation was found in cancer cells treated with metformin, restraining mTORC1 signaling and limiting the growth of renal cell cancer xenografts [52]. Frequent downregulation of AMPK has been observed in RCC and studies reported that metformin inhibits HIF1α stabilization [53] and activates AMPK leading PDL1 phosphorylation [21]. Consequently, metformin is thought to enhance anti-tumor CTL immunity by blocking the PDL1/PD1 axis.

In conclusion, our data demonstrated that sunitinib-resistant ccRCC cell lines aberrantly expressed PD-L1 and presented an increased activation of the Axl and MAPK pathways, and a metabolic shift toward oxidative phosphorylation and glutamine metabolism. Using a co-culture model with CTLs, we found that combining metformin with PD-L1 blocking therapeutic antibodies enhanced CTLs efficacy against sunitinib-resistant ccRCC cell lines. Therefore, combining metformin with PD-1/PD-L1 blocking therapies may have a potential value for ccRCC resistant to sunitinib.

## Supplementary Material

Supplemental Figure1.tif
