# Supplementary figures and images for: Impact of sunitinib resistance on clear cell renal cell carcinoma therapeutic sensitivity in vitro
Source: Cell Cycle. 2024 Jan 23;23(1):43–55. doi: 10.1080/15384101.2024.2306760 (PMC11005810; doi:10.1080/15384101.2024.2306760)

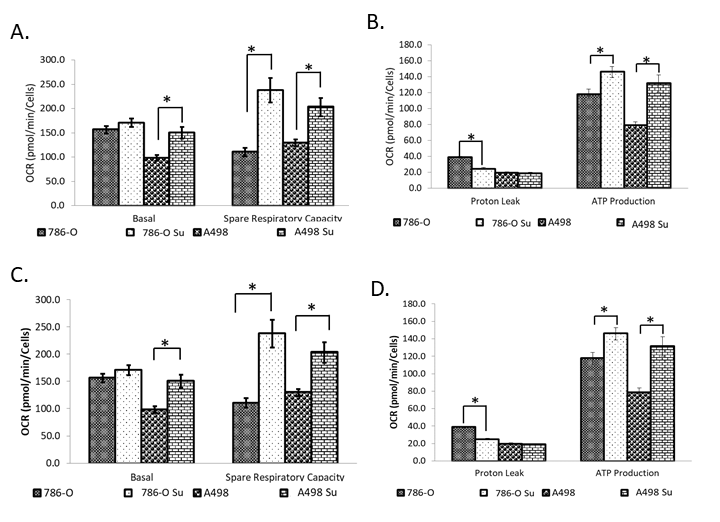

Supplement: Supplemental Figure1.tif [file KCCY_A_2306760_SM3782.tif]
